# 65 Candidozyma auris in the United States: Insights from the National Association of Long-Term Hospitals (NALTH) Network

**DOI:** 10.1017/ash.2026.10496

**Published:** 2026-06-23

**Authors:** Mateusz Gajda, Jadwiga Wókowska-Mach, Dariusz Hareza, Cezary Kapturkiewicz, Monika Pomorska-Weso Åowska, Dominika Kapustka

**Affiliations:** 1 Jagiellonian Univeristy / Univeristy Hospital in Cracow; 2 Jagiellonian University Medical School; 3 Northwestern University; 4 Analytical and Microbiological Laboratory of Ruda Slaska KORLAB

## Abstract

**Introduction** Urinary tract infections (UTIs) are among the most common infectious diseases and rank as the second most prevalent infection worldwide. Objectives This study aimed to identify the species-specific distribution of pathogens in positive UTI cultures from both hospitalized and ambulatory patients and to evaluate the applicability of current UTI treatment guidelines in patients from Silesia, southern Poland. Patients and Methods In this retrospective, multicenter study, 85,485 urine samples collected in Poland between 2022 and 2023 were analyzed. Samples were obtained from inpatients (n = 52,294) and outpatients (n = 33,191) with suspected UTIs. Participants were categorized into three groups: premenopausal women, postmenopausal women, and men. Antibiotics were classified using the WHO AWaRe (Access, Watch, Reserve) framework. Results Overall, antibiotic susceptibility was low. The estimated risk of ineffectiveness of empirically prescribed therapies ranged from 26.7% to 44.6%, depending on whether patients were treated as inpatients or outpatients. Among inpatients, Escherichia coli showed 73% susceptibility to co-trimoxazole and ?86.9% susceptibility to cephalosporins only in premenopausal women, compared with approximately 76.6% in men and postmenopausal women. Klebsiella pneumoniae demonstrated susceptibility exceeding 80% only to cefoperazone/sulbactam (86.3%), while susceptibility to other agents remained below 60%. In outpatients, E. coli isolates were highly susceptible to nitrofurantoin (91.9%); however, most other Access-group antibiotics showed susceptibility rates below 60%, except gentamicin (92%). Resistance mechanisms were common: extended-spectrum ?-lactamase production was detected in 12.8% of outpatient and 32.9% of inpatient Enterobacterales isolates, while high-level aminoglycoside resistance was observed in 56.5% of inpatient Enterococcus faecalis and 63.7% of Enterococcus faecium. Conclusion Given the substantial risk of empirical treatment failure under current guidelines, their alignment with real-world clinical practice should be reassessed using more extensive and locally derived epidemiological data.

